# Same Average, Different Odds: Maternal-health Lessons for India from Rwanda and Peru

**DOI:** 10.12688/f1000research.184633.1

**Published:** 2026-06-30

**Authors:** Shyamkumar Sriram

**Affiliations:** 1 Department of Rehabilitation and Health Services, University of North Texas College of Public Affairs and Health Sciences, Denton, Texas, USA

**Keywords:** maternal mortality; India; health equity; three delays; community health workers; financial protection; Rwanda; Peru

## Abstract

**Background:**

India has reduced its maternal mortality ratio faster than the global average, reaching 93 per 100,000 live births in 2019–21 and 88 in 2021–23. The national figure hides a nearly nine-fold gap between the best and worst-performing states. Deaths that remain fall mainly on rural, tribal, and poor women, in whom the three classic delays in obtaining care overlap with poverty, distance, caste, and gender.

**Policy and implications:**

The most useful lessons for India come not from wealthy systems but from countries that started poorer and still moved faster on equity. Rwanda combined a village health-worker cadre, near-universal community-based insurance, performance-based financing, and maternal death audits, working on the cost barrier and the second and third delays at once. Peru marginalized Andean and Amazonian women through free insurance, maternal waiting homes, and culturally adapted childbirth, working on first and second delays. Peru also shows that progress can be reversed and that judging a program only by its targets can erode the care it is meant to deliver.

**Recommendations:**

India should hold leadership of the gap between states rather than the national average, take the cost of delivery and its complications off poor families, fund respectful and culturally appropriate care alongside facility expansion, pay for audited quality, and track women’s experience of care, not coverage alone.

**Conclusions:**

India has shown that it can move its national numbers. The harder task is distributional: giving a woman in Assam or tribal Madhya Pradesh the odds of being a woman in Kerala. Rwanda and Peru suggested that this is within reach without a high-income budget, provided that the system is rebuilt around financial protection, a trusted community workforce, audited quality, and genuine respect for women.

## Introduction

India has done something remarkable with regard to maternal survival, and the headline numbers indicate this. The national maternal mortality ratio fell from 130 per 100,000 live births in 2014–16 to 93 in 2019–21, and the most recent bulletin puts it lower still, at 88 for 2021–23. ^1^ Facility birth has become the rule rather than the exception, reaching 88.6% of deliveries by 2019–21, with a skilled attendant present at a similar share. ^2^ Cash incentives and free public delivery have pulled millions of women in, and on its present path the country should pass the Sustainable Development Goal threshold of 70 ahead of 2030. A few would have predicted this two decades ago.

**Figure 1.**
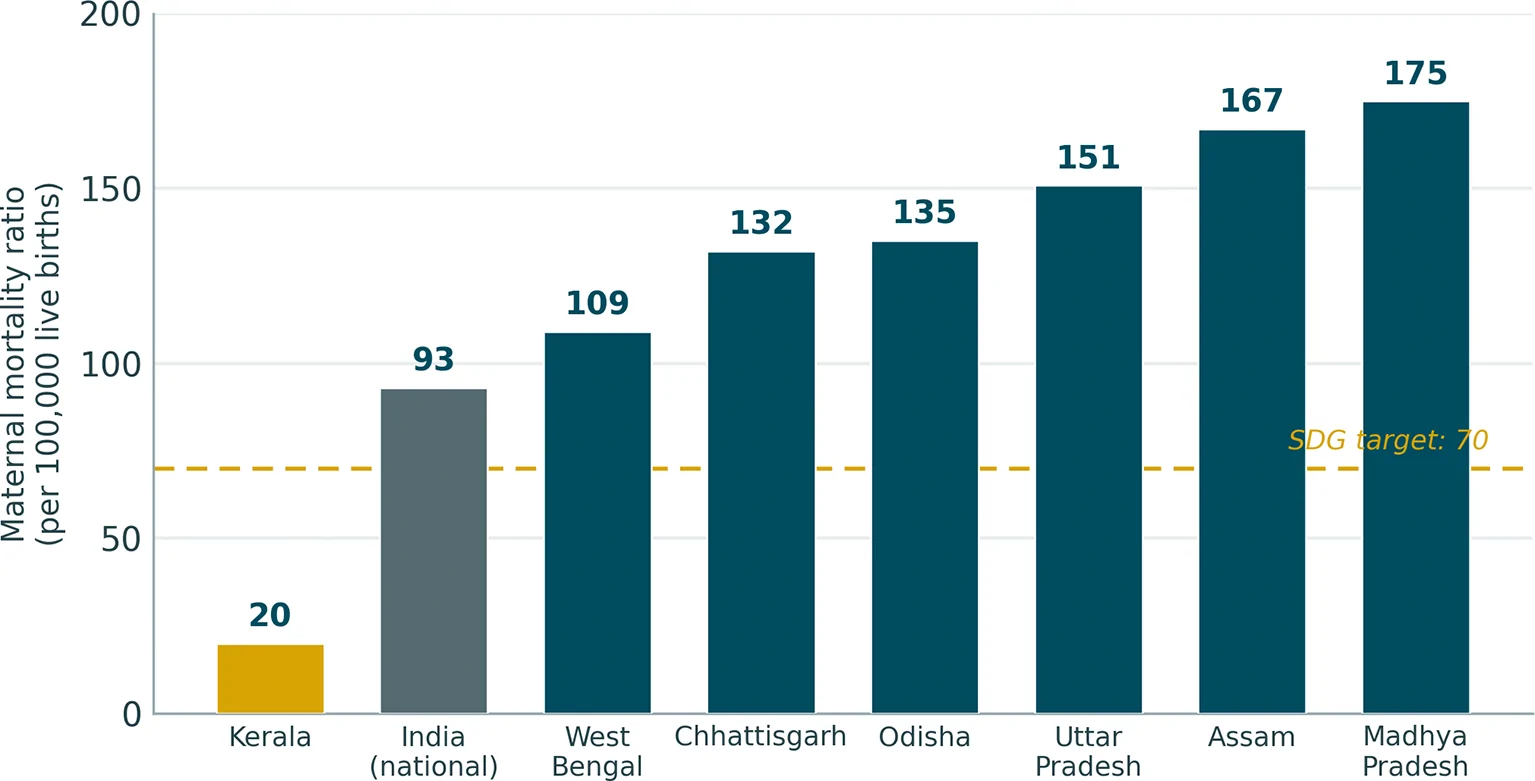
Maternal mortality ratio across selected Indian states, 2019–21. Maternal mortality ratio (deaths per 100,000 live births) for selected Indian states against the national figure and the Sustainable Development Goal target of 70, from the Sample Registration System Special Bulletin for 2019–21. Values span from Kerala (20) to Madhya Pradesh (175), a nearly ninefold range.

**Figure 2.**
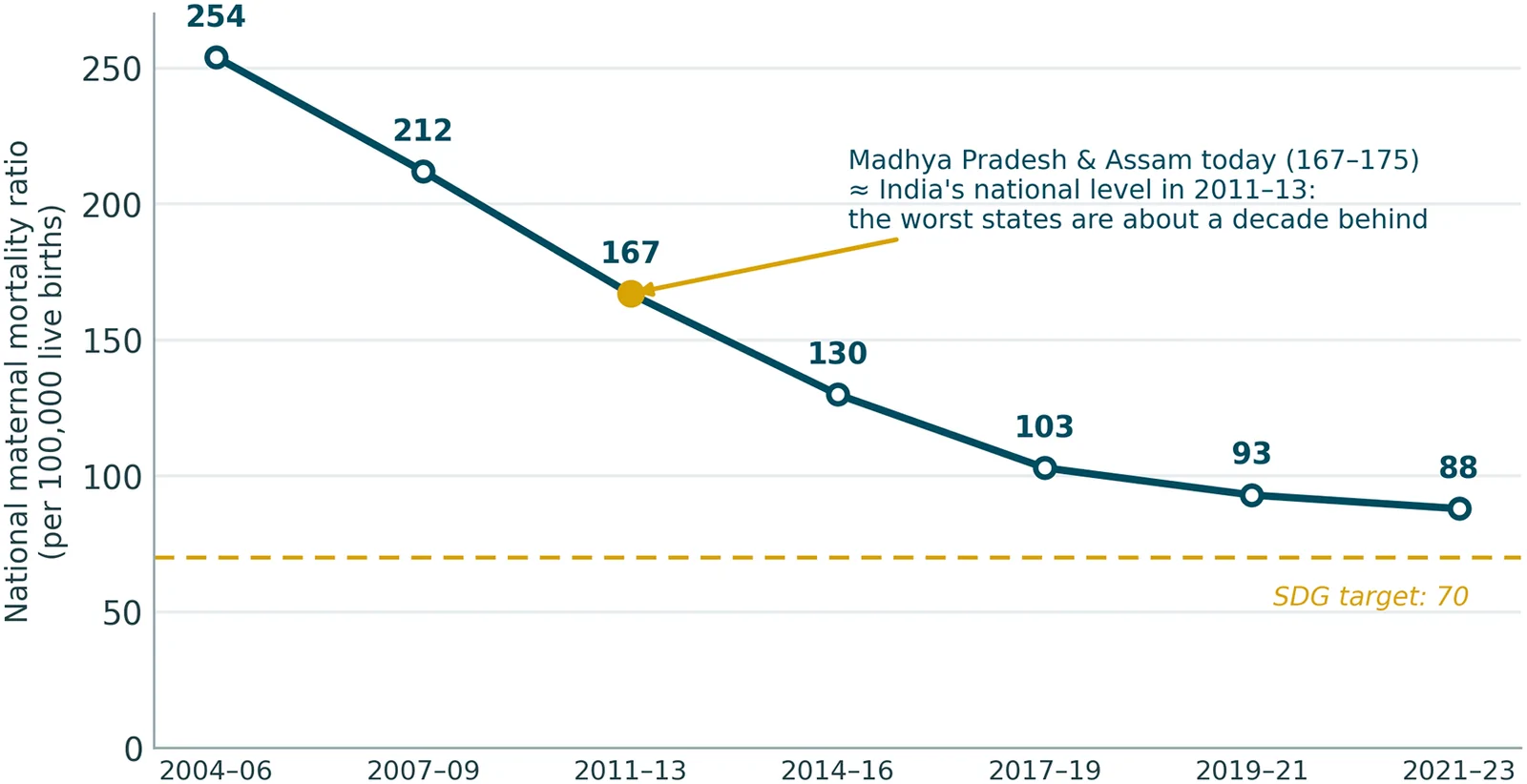
India’s national maternal mortality ratio across Sample Registration System rounds. National maternal mortality ratio (deaths per 100,000 live births) from 2004–06 to 2021–23 against the Sustainable Development Goal target of 70. The highlighted point marks 2011–13; Madhya Pradesh and Assam (167–175) remain at that level today, a lag of roughly a decade.

The national figure hides, as it shows. Kerala sits at 20, the kind of number you would expect in a rich country, while Madhya Pradesh and Assam are still at 175 and 167 ( Figure 1). ^1^ A woman’s chance of surviving childbirth depends heavily on which state she lives in. The same pattern holds over time. India’s national ratio has fallen steadily across successive Sample Registration System rounds, but the worst-performing states have not kept pace, and at 167 to 175 today Madhya Pradesh and Assam sit roughly where the country as a whole stood in 2011–13 ( Figure 2). ^1^ On this measure they are close to a decade behind.

Who dies and why completes the picture? The remaining deaths fall disproportionately in women who are rural, poor, tribal, and less schooled and occur in the three places Thaddeus and Maine identified 30 years ago: in deciding to seek care, in reaching a facility, and in getting adequate treatment once there. ^3^ These delays rarely arrive on their own. They stack on one another and on poverty, distance, caste, and gender, so a single woman is exposed to several things at once. It is this compounding of disadvantage, rather than any one barrier, that makes death difficult to prevent.

These numbers bear this out: Of the roughly 1.3 million maternal deaths India recorded over the two decades to 2020, close to two-thirds occurred in its poorer states, and obstetric hemorrhage, the most treatable of the major causes, accounted for almost half, more so in those same states. ^4^ A study of nearly two million pregnant women across the nine high-burden states put the local maternal mortality ratio at 383, far above the national figure, and found that women without any health-financing scheme were more than two and a half times as likely to die. ^5^ Scheduled-caste and scheduled-tribe women carried added risk. The same analysis offered a hopeful corollary: much of the danger attached to poverty fell away once a woman had reliable access to care during pregnancy and childbirth. This barrier is not destiny. It is design.

The money runs through all of these. Even with free public delivery and cash transfers, out-of-pocket spending on institutional birth has increased in most settings, and it regularly tips poor households into borrowing and distress sales. The caesarean rate, now near a fifth of births, runs at roughly twice the level clinical need would warrant and add to the bill. ^6^ So the binding constraint has shifted. Getting women into a facility is no longer the main problem: what happens once they arrive and whether the poorest reaches care at all? Antenatal care shows the same fault line: only about three in five women completed the four recommended visits, and fewer than one in five reached the eight World Health Organization advises, with the shortfall concentrated among poor and uninsured women. ^2^ India’s remaining maternal burden is therefore less a question of national capacity than of distribution, financial protection, acceptability, and the quality of care at the margins.

Where should India look for new ideas? The usual reflex is to benchmark against rich countries, but their budgets and staffing have remained out of reach for a decade or more. The better comparators were places that began poorer than India and still moved faster; above all on equity, India now struggles with. Approximately 260,000 women died of maternal causes worldwide in 2023, roughly one every two minutes, and the steepest falls have come from low- and middle-income countries that rebuilt how care reaches women rather than simply spending more. ^7^ Two of them, one in sub-Saharan Africa and one in Latin America, speak directly to India’s three delays.

## Policy outcomes and implications

### Rwanda: Financial protection, a community cadre and accountability

Rwanda entered this century as one of the poorest countries in the world and its health system in ruins after the 1994 genocide. Two decades on, its maternal mortality had dropped from over 1,000 per 100,000 live births around 2000 to roughly a quarter of that, and skilled attendance at birth had climbed from under a third to almost universal. ^8^ Asked what made the difference, district health managers kept returning to the same two answers: community health workers and health insurance. ^8^

How that worked is worth spelling out because each piece had an Indian echo. A cadre of village health workers, several to a community and answerable to the local health center, registers pregnancies and walks women through antenatal visits and into facility delivery, which goes straight during the first two delays. Community-based insurance, Mutuelles de Santé, with premiums scaled to household means and waived for the poorest, lifts the charge off the point of care, the charge that deters both women and ruins families. Rwanda did not stop getting women through the doors. It paid facilities partly on results, and in a cluster-randomized trial, performance-based financing raised institutional deliveries by 23% and improved the quality of antenatal care where payment followed performance. ^9^ Over the top of all these sit maternal death audits with senior oversight, so that a hemorrhage death becomes a reviewed event with a corrective action rather than a statistic.

For India, the lesson is less of a single instrument than the way Rwanda bolted them together. Financial protection, a trusted frontline workforce, and real accountability ran as one design and not as three schemes in separate lanes. India already has one version each. What it lacks is the joining-up, and accountability bites least in precisely the states where mortality is highest.

### Peru: Meeting women where they are

Peru’s problems are familiar to anyone who has worked in rural India. The women most likely to die were indigenous, poor, and remote, and they stayed away from facilities that felt foreign and treated them badly. Peru reduced maternal deaths substantially in the two decades before the pandemic, and it did so by rebuilding care around those women instead of waiting for them to fit the clinic.

Three measures were used in the majority of studies. Free public insurance, the Seguro Integral de Salud, began with poor pregnant women and children and pulled the previously excluded groups into care. ^10^ Maternal waiting homes, the casas de espera, let women from far-off communities stay near a working facility as their due date neared, which quietly dissolved the second delay of distance and transport. ^11^ And an intercultural birthing policy, written into a national technical norm, made room for upright delivery, the presence of family, and the use of Quechua or Aymara, so that a skilled, facility-based birth no longer felt like surrendering one’s culture. Together, these moved births out of homes and into facilities without asking women to abandon what childbirth meant to them, and skilled attendance climbed above nine in ten births. ^12^

### A caution: Gains can reverse, and targets can mislead

Peru carries two warnings worth taking on its board before borrowing. First, progress is not safe once won. COVID-19 knocked out Peru’s maternal mortality back to levels it had not seen before 2010, a blunt reminder that survival rests on services staying open and can unravel in a single shock. ^12^ The second is subtler. How do you measure how a programme changes what it has become? When health workers in the intercultural programs were judged only on facility births and deaths, some leaned on women to comply, and the respect for culture that the policy promised curdled into a form to be filled in. ^13^ The design was sound; collapsing it into a target was not. For a system such as India, which runs on dashboards, that is, the more awkward lesson, and probably the more important one. It also points to deeper caution: an instrument lifted out of the surrounding system tends to lose what made it work.

### What the two cases mean for India

Set side by side, Rwanda and Peru solved different halves of the same problem, and between them, they touched on every delay that still killed Indian women ( Table 1). Rwanda shows how to remove the cost from the equation and hold the system accountable for quality. Peru demonstrates how to make a facility both reachable and acceptable to women at the margins. Neither needed an Indian-sized budget. Both treated equity, not the average, as the point and worked because they went after the stacked disadvantages that gathered the last deaths among the poorest women. The levers are not just plausible on paper; several carry measured effects in the two countries ( Table 2).

**Table 1.** Three delays mapped to Rwandan and Peruvian levers and Indian gaps. The three delays framework (Thaddeus and Maine) set against the principal maternal-health levers used in Rwanda and Peru, with the corresponding Indian counterpart and gap.

Delay (Thaddeus & Maine)	Rwanda’s principal lever	Peru’s principal lever	Indian counterpart and gap
Delay 1: deciding to seek care	Village health workers who register pregnancies and bring women in for antenatal and facility care.	Culturally adapted childbirth (upright delivery, family presence, local languages) that makes facilities acceptable to indigenous women.	ASHAs do this work, but cultural acceptability and respectful care carry little programmatic weight, above all in tribal districts.
Delay 2: reaching a facility	A dense primary-care network built around health posts close to communities.	Maternal waiting homes (casas de espera) that house women near a facility before the due date.	Access has improved, yet remote and hilly blocks still face long transfers, and waiting homes are patchy.
Delay 3: receiving adequate care	Performance-based financing and maternal death audits that tie payment and oversight to quality.	Free insurance (SIS) that removes the point-of-care charge and funds skilled attendance.	Free public delivery exists, but out-of-pocket costs, referral failures and uneven emergency obstetric quality persist.

**Table 2.** Documented effects of signature maternal-health levers in Rwanda and Peru. Signature maternal-health levers in Rwanda and Peru, the delay each chiefly addresses, and the documented effect. Superscripts refer to the supporting sources in the reference list.

Country	Signature lever	Delay targeted	Documented effect
Rwanda	Community health-worker cadre	Delays 1–2	Skilled birth attendance rose from under a third to near-universal ^8^
Rwanda	Community-based insurance (Mutuelles de Santé)	Cost barrier	Over 90% of the population enrolled at peak coverage ^8^
Rwanda	Performance-based financing	Delay 3	A 23% rise in institutional deliveries in a cluster-randomised trial ^9^
Peru	Free maternal insurance (SIS)	Cost barrier	Higher access among the poorest, previously excluded groups ^10^
Peru	Maternal waiting homes (casas de espera)	Delay 2	Remote women brought within reach of skilled care ^11^
Peru	Intercultural, adapted childbirth	Delay 1	Skilled attendance lifted above nine in ten births ^12^

### India already holds most of the pieces

What makes this more than just an academic comparison is that India has not started from scratch. It has an accredited social health activist in nearly every village, a conditional cash transfer for institutional birth, a guarantee of free delivery and newborn care in public facilities, antenatal outreach days, a labor-room quality initiative, and a national scheme that covers hospital care for the poor. This resembles the Rwandan package. This gap lies in execution. Entitlements do not always add up to a zero bill for women who need a caesarean section in the morning; frontline workers are often stretched and lightly supervised; quality initiatives reach the better-run districts first; and maternal death reviews, where they happen at all, rarely change what the system does next. The high-burden states, where hemorrhage deaths and uninsured women gather, are where this machinery is the weakest. Closing the gap is less about inventing new schemes than about making the existing ones land together and land hardest, where the need is greatest.

## Actionable recommendations

They are ordered according to their priority. The first is foundational and the remainder reinforces it.

1. **Target the gap, not the average.** Make the spread between states rather than the national mean; figure leadership answers. Set firm and audit milestones for the worst-affected states and aspirational districts, report progress against them publicly, and steer extra staff, supervision, and money to where the stacked disadvantages sit rather than spreading it evenly.
2. **Take cost out of childbirth for poor women.** Make delivery and its complications, caesarean and emergency referral included, genuinely free at the point of care for poor households, and align the cash and hospital insurance schemes so that no family is driven into debt by birth. The finding that a missing financing scheme more than doubles a poor woman’s risk of dying ^5^ is sufficient to treat this as a clinical intervention and not as a welfare extra.
3. **Pay for care women will accept.** The investment in making facility birth welcomes tribal and remote communities, drawing on Peru’s intercultural model, and places maternal waiting homes only where a functioning emergency obstetric unit sits beside them, so that proximity buys real care and not merely a bed.
4. **Pay for quality, and make every death count.** Extend performance-linked payment to maternal services in the lagging states, tie it to managing the conditions that actually kill, hemorrhage above all, ^4^ and give maternal death surveillance real teeth so that each death produces a documented change in practice, as in Rwanda.
5. **Measure the experience of care, not only its coverage.** Track respect, consent, and what women actually go through alongside facility-birth counts and watch for the Peruvian failure mode, in which a target meant to help hardens into a stick. Coverage already appears good, and the next gains lie in concealed coverage.

## Conclusions

India has shown that it can shift its national maternal mortality rate. The work that remains is harder to do and harder to measure because it is about distribution: making the odds for a woman in Assam or in a tribal block of Madhya Pradesh look more like the odds for a woman in Kerala. Rwanda and Peru, both poorer than India when they began, suggest that this is within reach without waiting for a wealthy country’s budget, provided that the system is rebuilt around financial protection, a trusted community workforce, audited quality, and real respect for the women it is meant to serve.

These two precautions tempered the comparison. Rwanda and Peru are far smaller and more centrally governed than any large Indian state, and an instrument that worked in Kigali or the Peruvian sierra will not transfer unchanged to Bihar or the northeast, which lies in principles rather than templates. Cross-country figures rest on different data systems and baseline years; therefore, they describe direction and magnitude rather than a like-for-like ledger. What India needs next is not another national average but better disaggregated, district-level evidence on where deaths cluster and why, and implementation research that tests whether bundling financial protection, a strengthened frontline, and audited quality actually move outcomes in the highest-burden districts. What India needs most, in the end, is not new technology. However, this was a different target.

## Ethics and consent

Ethical approval and consent were not required.

## Data Availability

None of the data were included in this study. These figures were generated from publicly available published summary statistics, all of which are cited in the reference list. Figures were produced in Python using Matplotlib (version 3.10.8).
