# Supplementary material for: Same Average, Different Odds: Maternal-health Lessons for India from Rwanda and Peru
Source: F1000Res. 2026 Jun 30;15:1042. [Version 1] doi: 10.12688/f1000research.184633.1 (PMC13510846; doi:10.12688/f1000research.184633.1)
Supplement: Peer review history

# Reviewer report: Reviewer response for version 1

**DOI:** 10.5256/f1000research.203797.r501870

**Published:** 2026-07-24

**Authors:** Jang Bahadur Prasad

**Affiliations:** 1 Jawaharlal Institute of Postgraduate Medical Education & Research, Puducherry, Puducherry, India

Overall Assessment

 This manuscript provides a concise and well-reasoned policy analysis that examines how experiences from Rwanda and Peru may inform strategies to reduce maternal mortality disparities in India. Rather than presenting another descriptive review of maternal mortality, the author develops a clear comparative argument centered on health system design and equity. The manuscript makes a useful contribution by demonstrating that successful maternal health policies cannot be understood as isolated interventions but as coordinated system-level approaches. This perspective distinguishes the article from many existing policy commentaries.

 A particular strength is the balanced treatment of policy transfer. The manuscript avoids suggesting that interventions implemented elsewhere can simply be replicated in India and instead acknowledges contextual differences and the need for adaptation. This nuanced interpretation strengthens the credibility of the analysis.

 Strengths

 The manuscript is built around a clear conceptual framework based on the "three delays" model, which provides a logical structure for comparing the experiences of Rwanda and Peru with India's current maternal health challenges.

 The synthesis of evidence is selective and focused. Rather than attempting an exhaustive literature review, the manuscript integrates the most relevant evidence into a coherent policy argument.

 The comparison between the two countries is complementary rather than repetitive. Rwanda illustrates system accountability and financial protection, whereas Peru highlights culturally responsive service delivery and geographic accessibility.

 The discussion appropriately recognizes that reducing maternal mortality requires improving quality, financial protection, and women's experiences simultaneously, rather than concentrating solely on increasing institutional deliveries.

 The manuscript concludes with practical recommendations that follow logically from the preceding discussion and remain appropriately cautious about generalizing across different health system contexts.

 Minor Comments

 Although the manuscript is clearly presented as a policy analysis, it would benefit from explicitly stating this objective in the Introduction. Doing so would better orient readers regarding the intended scope and methodology.

 The discussion could briefly acknowledge that evidence from Rwanda and Peru originates from health systems that differ substantially from India in population size and administrative complexity. While this limitation is mentioned later, introducing it earlier would improve the overall framing of the comparison.

 Some of the transitions between sections could be strengthened to improve continuity. In particular, a short linking paragraph between the country case studies and the synthesis section ("What the two cases mean for India") would enhance the flow of the manuscript.

 The article would benefit from a brief statement identifying priorities for future comparative implementation research, particularly studies evaluating how combinations of interventions perform in diverse Indian state contexts.

 The figures and summary tables effectively support the narrative and are easy to interpret. Only minor editorial refinement of captions for consistency with the main text may be considered.

 Recommendation: Accept

 This manuscript offers a well-constructed and policy-relevant comparative analysis that will be of interest to readers working in maternal health, health systems research, and public policy. The manuscript is clearly written, supported by appropriate evidence, and makes a meaningful contribution to the literature on maternal health equity. The comments above are intended only to further strengthen an already strong manuscript and do not require substantive revision. I recommend the manuscript for acceptance.

Does the paper provide a comprehensive overview of the policy and the context of its implementation in a way which is accessible to a general reader?

Yes

Is the discussion on the implications clearly and accurately presented and does it cite the current literature?

Yes

Are the recommendations made clear, balanced, and justified on the basis of the presented arguments?

Yes

Reviewer Expertise:

Biostatistics

I confirm that I have read this submission and believe that I have an appropriate level of expertise to confirm that it is of an acceptable scientific standard.

# Reviewer report: Reviewer response for version 1

**DOI:** 10.5256/f1000research.203797.r501869

**Published:** 2026-07-20

**Authors:** Rajeshwari Biradar

**Affiliations:** 1 School of Public Health, Sri Balaji Vidhyapeeth University, Bahour Taluk, Puducherry, India

**Overall assessment**

 This is a well-written, timely, and policy-relevant article that addresses an important public health challenge: reducing inequities in maternal mortality in India through lessons drawn from Rwanda and Peru. The manuscript is clearly structured, logically argued, and succeeds in translating international evidence into practical policy recommendations for India. The comparative approach is thoughtful and appropriately focused on health systems that have achieved substantial improvements despite limited resources, making the lessons highly relevant for low- and middle-income country settings.

**Major strengths**

 The manuscript presents a compelling narrative that shifts attention from national maternal mortality averages to persistent subnational inequities, which is an important and policy-relevant perspective.

 The comparison between Rwanda and Peru is well justified and provides complementary examples addressing financial protection, community health systems, culturally appropriate care, accountability, and quality improvement.

 The article effectively integrates evidence from published literature into a coherent policy discussion rather than simply describing interventions.

 The recommendations are practical, prioritized, and clearly linked to the evidence presented throughout the manuscript.

 The manuscript is highly readable. The writing style is engaging, concise, and accessible to both researchers and policymakers while maintaining scientific rigor.

 The inclusion of the limitations of cross-country comparisons strengthens the credibility and balance of the discussion.

**Minor comments**

 While the discussion appropriately emphasizes Rwanda and Peru, a brief acknowledgment of other successful maternal health experiences from comparable LMICs (e.g., Sri Lanka or Bangladesh) would further justify the selection of these two countries.

 Some policy recommendations could briefly mention potential implementation challenges within India's federal health system, particularly differences in governance capacity across states.

 Although the manuscript discusses respectful maternity care, a brief mention of existing WHO frameworks or quality-of-care indicators could strengthen this section.

 A few sentences throughout the manuscript are relatively long and could be divided to improve readability without changing the content.

 The conclusion could include one or two sentences highlighting future implementation research priorities or evaluation frameworks for assessing the proposed policy adaptations in India.

**Recommendation: Accept**

 This manuscript makes a valuable contribution to the maternal health policy literature. It provides an insightful and well-supported comparative analysis with clear implications for reducing maternal mortality inequities in India. The suggested revisions are minor and primarily intended to further strengthen the clarity and contextualization of an already high-quality manuscript. I recommend acceptance.

Does the paper provide a comprehensive overview of the policy and the context of its implementation in a way which is accessible to a general reader?

Yes

Is the discussion on the implications clearly and accurately presented and does it cite the current literature?

Yes

Are the recommendations made clear, balanced, and justified on the basis of the presented arguments?

Yes

Reviewer Expertise:

Demography and Public health

I confirm that I have read this submission and believe that I have an appropriate level of expertise to confirm that it is of an acceptable scientific standard.
